# Human Babesiosis, Yucatán State, Mexico, 2015

**DOI:** 10.3201/eid2411.170512

**Published:** 2018-11

**Authors:** Gaspar Peniche-Lara, Lucero Balmaceda, Carlos Perez-Osorio, Claudia Munoz-Zanzi

**Affiliations:** Universidad Autónoma de Yucatán, Yucatán, Mexico (G. Peniche-Lara, L. Balmaceda, C. Perez-Osorio);; University of Minnesota, Minneapolis, Minnesota, USA (C. Munoz-Zanzi)

**Keywords:** Babesia microti, tick-borne diseases, babesiosis, Mexico, vector-borne infections, protozoa, parasites

## Abstract

In 2015, we detected clinical cases of babesiosis caused by *Babesia microti* in Yucatán State, Mexico. Cases occurred in 4 children from a small town who became ill during the same month. Diagnosis was confirmed using conventional PCR followed by sequencing of the DNA fragment obtained.

Protozoa from the genus *Babesia*, specifically *B. microti* and *B. divergens*, can infect humans (*1*). Symptoms of infection include fever (38°C–40°C), malaise, chills, myalgia, and fatigue. In some cases, nausea, emesis, night sweats, weight loss, anemia, and hematuria are reported. Hepatomegaly and splenomegaly also can be present (*1*). Transmission occurs mainly when infected ticks of the *Ixodes* genus feed on humans; however, blood transfusions have been reported as another route.

In Mexico, the only report of human babesiosis is that of isolation of *Babesia* spp. from a blood sample from an asymptomatic human in 1976 (*2*). In Yucatán State, human cases of dengue fever and rickettsiosis are frequent and usually share similar symptoms to babesiosis, which is difficult to diagnose (*3*,*4*). We describe 4 patients with confirmed babesiosis caused by *B. microti* in Yucatán, Mexico.

## Case Reports

All 4 patients were from a small municipality in eastern Yucatán and had disease onset during July 2015. All patients lived in close proximity but in different households. After dengue fever was excluded at the local rural health center, blood samples were sent to the Parasitic and Infectious Diseases Laboratory at the Medicine Faculty of the University Autonomous (Yucatán, Mexico) for rickettsiosis diagnosis. Molecular test and immunofluorescence indirect assay for *Rickettsia* infection were negative in all samples. Independent from clinical management, we conducted additional testing on stored samples, and on the basis of the common history of risk factors, ticks, and rodent presence, we suspected babesiosis. Deidentified medical record information was limited about history, clinical presentation, and treatment.

Patient 1 was a 13-year-old boy who was seen at the health service center with signs and symptoms that included fever (39°C), myalgia, fatigue, and arthralgia that started 3 days before his clinic visit. His mother indicated he had frequent contact with vegetation because of work in agricultural activities and close contact with dogs, cats, birds, and cows and noted that ticks parasitized domestic animals and rodents surrounding the house.

Patient 2 was a 12-year-old girl who had a history of fever (38°C) during the previous 3 days that was unresponsive to medication, myalgia, fatigue, nonsevere arthralgia, drowsiness, and paleness. On the day of her clinic visit, myalgia was severe. Fever was initially treated at home with medicinal plants and later with paracetamol (500 mg/6 h). She lived with her parents and performed housework that included close contact with dogs and goats.

Patient 3 was a 14-year old male student with 14 days of symptoms that included fever (38°C), myalgia, arthralgia, and headache. Four days after symptom onset, he went to a private clinic and began treatment with amoxicillin and ambroxol but did not recover. On day 14, fever and other symptoms persisted, in addition to weight loss and drowsiness. He mentioned observing ticks feeding on backyard dogs and having close contact with vegetation while gardening at school.

Patient 4 was an 8-year-old girl with 3 days of fever (39°C), headache, drowsiness, and exanthema in the upper extremities. During physical examination, 2 ticks were found attached to her, 1 on the right groin area and 1 on the scalp. Ticks were not available for laboratory analysis. Her mother stated close contact with dogs in the house and presence of rodents.

For all blood samples, DNA was purified using Quick-gDNA MiniPrep (Zymo Research, Irvine, CA, USA). We used conventional PCR to amplify a 474-bp gene fragment from 18S rRNA of *Babesia* species (*5*). Amplified products were cloned and sequenced (GenBank accession no. MH048838). The amplified products were 100% homologus to *B. microti* from several isolates from Asia (GenBank accession nos. KF410826.1, LC005763.1, LC005772.1). Proper laboratory quality control measures included negative (sterile water as a sample) and positive (*B. microti* DNA sample) controls. We used separate areas and personnel for DNA extraction, quantitative PCR, and PCR reaction setup.

Because of the mild-to-moderate infection, all patents were treated with chloroquine. All progressed well during the treatment, and they completely recovered.

## Conclusions

Our findings document the occurrence of human babesiosis confirmed by molecular methods in Yucatán. Babesiosis is a widespread vectorborne disease transmitted by tick bites, which has similar clinical presentation to infectious diseases that are endemic to Yucatán, such as dengue and rickettsiosis. Increased awareness is needed among health providers about inclusion of babesiosis in the differential diagnosis of undefined febrile illness and to develop guidelines for timely diagnosis in adults and children. Furthermore, studies are required to evaluate the adaptation of *B. microti* to hosts and vectors to which it is endemic, characterize its clinical manifestations, and estimate the real prevalence and incidence of *Babesia* infection in the population of Mexico.
